# Aggression in children with behavioural/emotional difficulties: seeing aggression on television and video games

**DOI:** 10.1186/s12888-014-0287-7

**Published:** 2014-11-18

**Authors:** Oana Mitrofan, Moli Paul, Scott Weich, Nicholas Spencer

**Affiliations:** Academic Clinical Fellow, Warwick Medical School, University of Warwick, Gibbet Hill Road, Coventry, CV4 7AL UK

**Keywords:** Child, Aggression, Behavioural and emotional difficulties, Television, Video games

## Abstract

**Background:**

Mental health professionals are often asked to give advice about managing children’s aggression. Good quality evidence on contributory environmental factors such as seeing aggression on television and in video games is relatively lacking, although societal and professional concerns are high. This study investigated possible associations between seeing aggression in such media and the aggressive behaviour of children attending specialist outpatient child and adolescent mental health services (CAMHS).

**Methods:**

In this mixed methods study, forty-seven British children aged 7–11 years with behavioural/emotional difficulties attending CAMHS and their carers participated in a survey; twenty purposively-selected children and a parent/carer of theirs participated in a qualitative study, involving semi-structured interviews, analysed using the Framework Analysis Approach; findings were integrated.

**Results:**

Children attending CAMHS exhibit clinically significant aggression, of varying types and frequency. They see aggression in multiple real and virtual settings. Verbal aggression was often seen, frequently exhibited and strongly associated with poor peer relationships and low prosocial behaviour. Children did not think seeing aggression influences their own behaviour but believed it influences others. Carers regarded aggression as resulting from a combination of inner and environmental factors and seeing aggression in real-life as having more impact than television/video games.

**Conclusions:**

There is yet no definitive evidence for or against a direct relationship between aggression seen in the media and aggression in children with behavioural/emotional difficulties. Future research should take an ecological perspective, investigating individual, developmental and environmental factors. Carers, professional organisations and policy makers should address aggression seen in all relevant area of children’s lives, primarily real-life and secondly virtual environments.

## Background

Aggression and violence among children and adolescents are of worldwide public health importance [1]. Aggression is a common reason for referrals to child and adolescent mental health services (CAMHS) [2]. Referred children may have higher frequency and severity of aggression compared with non-referred peers [3].

CAMHS professionals are often asked to give advice about managing aggression in children, including psycho-education about contributory environmental factors. Possible associations between exposure to violence in the media and aggression, especially in younger children, have raised public health concern [4]. The impact on violent media on aggressive behaviour has been much debated, and the methodological quality of many studies and some early meta-analyses extensively criticised. Methodological problems, such as the use of non-standardised measures of aggression that were not tested for validity or reliability, the use of *proxy* measures of aggression that involved no direct physical aggression or violent behaviour, the lack of controlling for other factors and publication bias effects mean that the evidence remains inconclusive [5,6]. Recent meta-analyses indicate small overall effects for exposure to violence in passive media such as television (TV) and film and newer, interactive media such as video games on aggressive behaviour in children and adults (effect sizes range between r = .03 and .20, with a corrected effect size of r = .08 for children) [5-7].

Authors have suggested that research should focus on children at increased risk for aggression rather than the general population [4,8]. Children with pre-existing mental health problems, such as behavioural and emotional difficulties, (BED) were reported to be more susceptible to watching and/or being affected by media violence [4,9]. A systematic review focused on children with BED, however, found insufficient, contradictory and methodologically flawed evidence on such an association [10]. Most studies were North American and school-based; the few health-based studies focused on psychiatric diagnoses associated with aggression, such as conduct disorder, but not aggression *per se*. Yet, aggression is a non-specific behaviour commonly associated with various, but not equivalent to any, psychiatric diagnoses; aggression can be objectively measured and targeted for intervention, regardless of any associated diagnoses [3]. Levels of aggression in children with BED attending CAMHS are unknown. Frequencies of other factors that may account for or explain any observed relationship between seeing aggression in the media and aggressive behaviour (so-called third variables) are also unsubstantiated.

Research has been hindered by the lack of valid and reliable measures of seeing aggression in TV programmes and video games, the challenges of operationalizing various definitions of aggression and separating it from concepts such as violence and antisocial behaviour [2,10]. This paper focuses on direct or overt, other-directed aggression because of its high internal and external validity [2]. Overt aggression has two categories of physical and non-physical aggression, the latter encompassing verbal (e.g., saying hurtful things to another individual), symbolic (i.e., attempting to hurt an individual in a non-verbal manner e.g. making threatening gestures) and object (e.g., hitting an object) aggression [10]. The evidence base could also be improved by having children’s, potentially different, perspectives in addition to information from carers and professionals [11,12].

This complexity and numerous gaps in knowledge, especially about primary school-aged children, prompted this mixed-methods study of 7-11-year olds, aimed at better understanding possible associations between aggression in children with BED attending CAMHS and their seeing aggression in TV programmes and video games. The research questions were: What are the frequency and characteristics of children’s aggression? Where do children see aggression in their lives? What are the children’s and carers’ perspectives on associations between aggression seen in TV programmes and video games and children’s aggression?

## Methods

We conducted a survey on aggressive behaviour of children attending CAMHS, a qualitative study on the views of some of these children and their carers on where children saw aggression and possible associations between seen and exhibited aggression, and integrated the findings. The study was conducted in four specialist, multi-disciplinary, outpatient CAMHS in Coventry and Warwickshire. People in this mixed urban and rural area of the United Kingdom (UK) are broadly representative of the general UK population [13].

### Participants

Survey participants were recruited from all children referred to participating CAMHS, between November 2006 and May 2008, who met the following inclusion criteria: referred for behavioural difficulties (e.g., disruptive/challenging/aggressive/antisocial behaviour, hyperactivity, conduct problems) and/or emotional symptoms (e.g., depression, anxiety, withdrawal); aged between 7–11 years at referral. Children with generalised learning difficulties, psychoses, pervasive developmental, eating and substance-related disorders were excluded because of potentially different associations of aggression in such conditions. Other exclusion criteria included: having sensory impairments preventing TV/video game use; contemporaneous child protection issues; and non-English speakers.

Thirty-nine (17%) of the 226 eligible children and 47 (21%) of their main carers participated in the quantitative study. Age ranged between 7–11 years (mean 9 years, SD 1.4) at referral and 8–12 years (mean 10.2, SD 1.4) at time of participation. Almost 3/4 of the children were boys (*n* = 35; 75%); all but one were of White British ethnicity. Emotional problems were the most common reasons for CAMHS referral (*n* = 22; 47%), followed by non-specific behavioural (*n* = 10; 21%), hyperkinetic (*n* = 8, 17%) and conduct (*n* = 7, 15%) problems. All children watched TV (*n* = 47, 100%), most played video games on a console (*n* = 42, 89%) or handheld games (*n* = 38, 81%) and used computers (*n* = 40, 85%), mobile phones (*n* = 34, 72%) and the Internet (*n* = 38, 81%).

Qualitative study participants were purposively sampled for varying levels of aggression and difference in age, gender, ethnicity and family income [14]. Fifteen boys and five girls aged 8–12 years, and their main carers contributed to forty interviews, a number likely to deliver saturation of themes and facilitate triangulation of data (child interviews, carer interviews and quantitative data) [15]. All children but one were of White British ethnicity and had a wide range of family income.

The study was approved by a local research ethics committee. Permission for children’s participation was sought from the child (verbal assent) and a parent with Parental Responsibility (consent). Carers gave consent for their own participation.

### Measures

Carers completed the Children’s Aggression Scale, parent version (CAS-P), a measure of type, frequency and severity of aggression in psychiatrically referred children aged 7–11 years, in outpatient settings [16]. Most of its 33 items are rated on a 5-point severity/frequency scale (from “never” = 0 to “most days” or “more than 10 times” = 4). The CAS-P has 5 subscales: Verbal Aggression (e.g., from “snapped or yelled” to “verbally threatened to hit” others), Aggression Against Objects and Animals (e.g., from “slammed a door when angry” to “tortured a pet”), Provoked Physical Aggression (e.g., fighting with others when provoked resulting in mild to serious injuries), Initiated Physical Aggression (e.g., starting fights resulting in mild to serious injuries) and Use of Weapons (e.g., from carrying to injuring another with a weapon). Summing the products of the frequency of behaviour by severity weight for each item yields a total score: higher scores indicate greater aggression. With good internal reliability, the CAS-P significantly correlates with ratings of aggression on the Inattention/Overactivity with Aggression Scale and Child Behavior Checklist (CBCL) [16].

Children and carers completed the Measure of Aggression, Violence, and Rage in Children (MAVRIC), a measure of frequency and severity of aggression in children aged 5–18 years, in psychiatric outpatient/inpatient settings [17]. The 19 items on the MAVRIC-C (child version), which parallel those on the MAVRIC-P (parent version), contain between one and eight yes-no questions each, covering verbal and physical aggression and aggression against objects. Higher scores are assigned to “yes” answers indicating longer history and duration of aggressive outbursts and greater severity of potential harm to others. Items are summed to yield a total score (0–30). MAVRIC has good internal reliability and convergent validity with the Aggressive Behavior subscale of the CBCL [17]. A clinical cut-off of 10 was used [17,18]. The MAVRIC-C was read and explained to children, ensuring understanding.

Carers completed the standardised, validated and reliable Strengths and Difficulties Questionnaire (parent version, P4-16-SDQ), a brief behavioural screening measure of children aged 4–16 years [19]. The 25 items, each rated on a 3-point scale, are allocated to five subscales, generating scores (0–10) for Emotional Symptoms, Conduct Problems, Hyperactivity, Peer Problems and Prosocial Behaviour. Summing all but the last generates a Total Difficulties Score (0–40).

Semistructured, individual interviews explored children’s and carers’ views on the nature of aggression and where children see aggression in their lives, their feelings on seeing aggression, any relationship between seeing aggression and behaving aggressively and any influencing factors. Children were first asked about TV programmes/video games they liked/did not like. Probing questions, using cartoons, explored programme/game content, what the “goodies” and “baddies” did, whether anything from the programmes/games scared them, and any programmes/games they were not allowed to watch/play. Children were then shown a set of pictures illustrating aggression and asked to describe what they thought was happening in each picture, whether and where they previously had seen such things happening and how they felt at the time, and whether children, including themselves, do such things after seeing them. Most pictures were taken from the Violence Exposure Scale-Revised [20]. Carers were asked similar open-ended questions.

### Procedure

All children’s study measures were completed on the same day, either at CAMHS or the child’s home, either with the child alone (carer in a room nearby) or in the carer’s presence (as facilitator). The interviews took approximately 40 minutes (10 minutes for the MAVRIC-C). Carers’ interviews were up to 90 minutes long (including approximately 5–10 minutes per questionnaire), completed on the same or later date, depending on carers’ availability. Interviews were audiotaped and transcribed verbatim. Respondent validation for researcher interpretation was sought during interviews.

### Data analysis

Descriptive statistics were compiled frequency and characteristics of aggression. Spearman’s correlation and group comparisons using Kruskal-Wallis, Mann–Whitney U tests and Wilcoxon test for matched samples were used to examine associations between scores on aggression measures, socio-demographic variables and SDQ scores. The child’s age at referral was used in statistical analyses as this was a sampling criterion.

Interview transcripts were analysed using the five-staged Framework Approach, a form of thematic qualitative analysis [21], and NVivo software (version 8). The central component of the Framework Approach is the thematic framework, a series of thematic headings sorted into main and sub-themes, generated from anticipated and emerging issues. The framework is systematically applied to every transcript. The analyst moves back and forth between levels of abstraction without losing sight of the original data, ensuring consistency and transparency. The full range of views can be compared and contrasted within and across participants, thus patterns can be identified and explored.

The quantitative analysis preceded and facilitated qualitative analysis. Issues identified through qualitative analysis informed further quantitative analysis, e.g. exploring links between aggression and the child’s age and family income.

## Results

### Frequency and characteristics of children’s aggression

Children exhibited various types of aggression: aggression against objects and animals, verbal and physical aggression, including severe forms e.g., attempting to kill someone. Mean CAS-P scores, reflecting frequency and severity of aggression, ranged from 0.43 (weapon use) to 8.83 (verbal aggression) (Table 1). MAVRIC mean scores were above the cut-off of 10 (child report 14.59, SD 5.34, range 2–23; carer report 14.65, SD 5.53, range 3–26), suggesting clinically significant aggression in this population. About three-quarters of children (*n* = 28, 72% on self-report; *n* = 36, 78% on carer report) scored above this cut-off. Eleven children (28%) reported thoughts of killing other people when angry and three (8%) having tried to do so. Seventeen carers (37%) reported their children having such thoughts, while three (6%) reported their children’s attempts. Two carers (6%) reported gang-related weapon use. Carer reports were strongly associated with each other (ρ ranging from 0.52 to 0.72, *p* < .05), but not with child reports.

**Table 1:** **Means and standard deviations (SD) of scores on aggression measures for total sample and by socio-demographic characteristics**

	**MAVRIC-C**	**MAVRIC-P**	**CAS-P verbal**	**CAS-P objects & animals**	**CAS-P provoked physical**	**CAS-P initiated physical**	**CAS-P weapons**	**CAS-P total score**
Total	14.59	14.65	8.83	2.36	2.99	2.32	0.43	17.06
	(5.34)	(5.53)	(5.33)	(1.8)	(2.49)	(2.23)	(1.29)	(11.16)
Age group	13.63	15.79	9.29	2.72*	3.12	2.68**	0.62	18.7
7–9 years	(5.76)	(5.2)	(4.96)	(1.69)	(2.38)	(2.16)	(1.63)	(10.31)
Age group	16.13	12.89	8.13	1.79*	2.78	1.76**	0.14	14.6
10–11 years	(4.32)	(5.72)	(5.95)	(1.87)	(2.71)	(2.28)	(0.33)	(12.22)
Below NAI	14.88	16*	10.13*	2.84*	3.61*	2.55	0.64*	19.79*
	(5.8)	(5.23)	(5.83)	(1.99)	(2.67)	(2.45)	(1.54)	(12.11)
Above NAI	14.9	11.93*	6.28*	1.48*	1.83*	2	0*	11.6*
	(3.9)	(5.66)	(3.38)	(0.93)	(1.72)	(1.82)	(0)	(6.6)

MAVRIC = Measure of Aggression, Violence and Rage in Children (C = child version; P = parent version); CAS-P = Children’s Aggression Scale, parent version; NAI = national average income.

*p < .05 **p < .10.

Several socio-demographic factors were associated with children’s aggression: age, average family income, household size (number of people living in the home), main carer’s highest level of formal education and household type (family headed by lone parent or couple). Seven-nine year-olds scored higher than 10–11 year-olds on Aggression against Objects and Animals, with a similar trend for Initiated Physical Aggression. Children from below national-average family income homes scored higher than others on most aggression measures (Table 1). Children in the lowest income group (mean 0.96, SD 1.88) scored higher than others (mean 0.04, SD 0.14) on Use of Weapons (*p* < .05). Larger household size was linked to high scores on overall CAS-P, Verbal Aggression and Initiated Physical Aggression (Table 2). There was a trend for children whose carers’ highest level of formal education was secondary school (mean 0.54, SD 0.97), living in a family headed by a lone parent (mean 1.34, SD 2.47) to score higher on Use of Weapons than children whose carers’ highest level of formal education was university (mean 0, SD 0; *p* < .10), living in a family headed by a couple (mean 0.17, SD 0.47; *p* < .10). Aggression scores were not linked to gender.

**Table 2:** **Correlations between aggression scores, household size and SDQ scores**

	**Household size**	**SDQ conduct problems**	**SDQ peer problems**	**SDQ prosocial behaviour**
MAVRIC-C	−0.04	0.3	0.42*	−0.21
MAVRIC-P	0.19	0.23	0.38*	−0.48*
CAS-P Verbal	0.33*	0.53*	0.36*	−0.6*
CAS-P Objects & Animals	0.25	0.4*	0.32*	−0.57*
CAS-P Provoked Physical	0.19	0.2	0.24	−0.37*
CAS-P Initiated Physical	0.4*	0.23	0.16	−0.36*
CAS-P Weapons	−0.09	0.23	0.43*	−0.53*
CAS-P total score	0.32*	0.47*	0.31*	−0.6*

*Note:* MAVRIC = Measure of Aggression, Violence and Rage in Children (C = child version; P = parent version); CAS-P = Children’s Aggression Scale, parent version; SDQ = Strengths and Difficulties Questionnaire.

*p < .05.

High aggression scores on most subscales were associated with high levels of peer relationship problems and low prosocial behaviour levels on the SDQ. High Verbal Aggression scores were more strongly linked to high Conduct Problems and Peer Problems and low Prosocial Behaviour SDQ scores than most of the other aggression subtypes (Table 2).

### Where do children see aggression?

**Figure 1:**
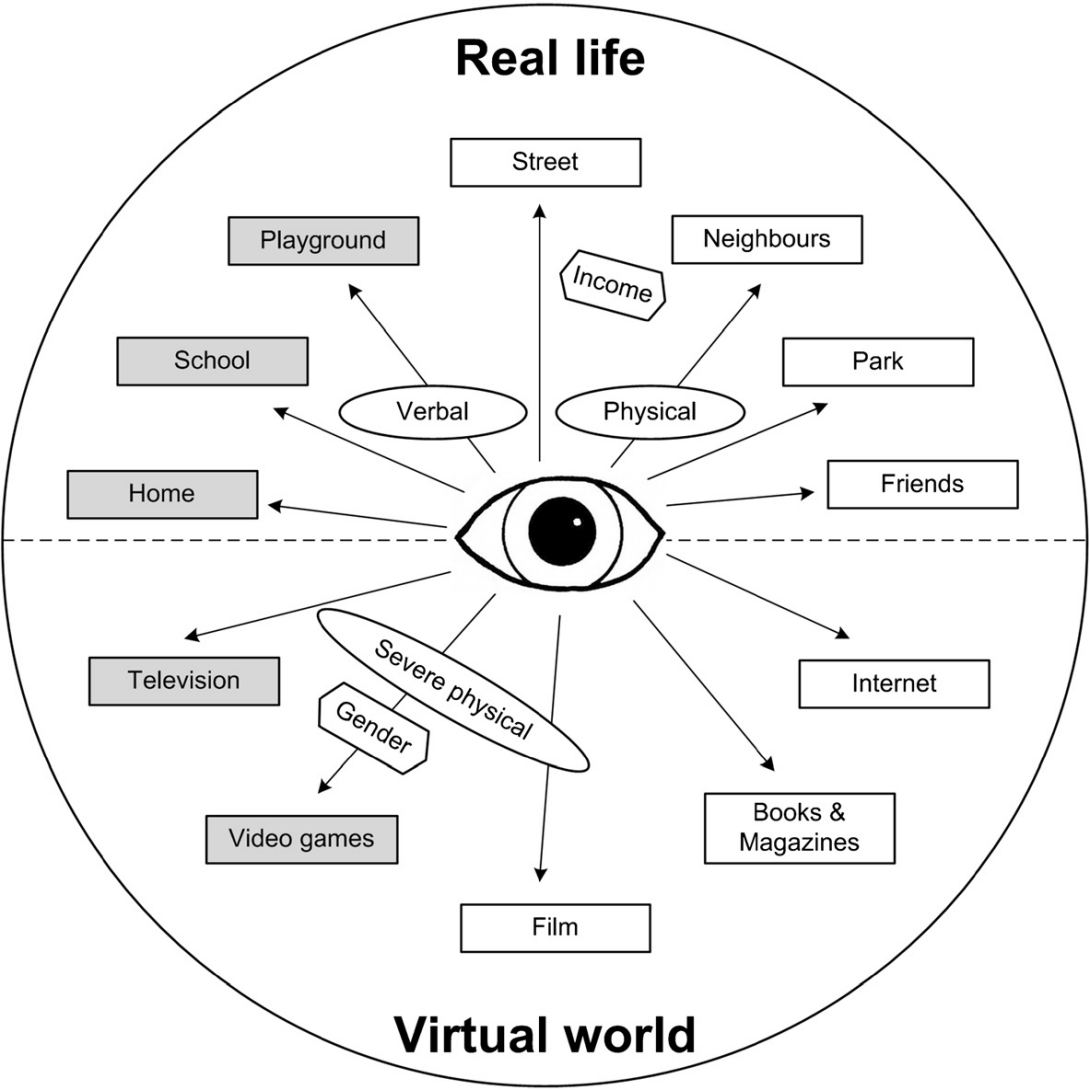
**Where do children see aggression in their lives.** Sources of seeing aggression. Main sources of seeing aggression. Main types of aggression. Important factors related to sources of seeing aggression.

Children and carers described many places where children see aggression. They fall into two broad categories: real-life, mainly school, playground and home; and the virtual world, mainly television and video games (Figure 1). Video games and TV programmes were the most often reported sources of seeing aggression.

Real-life aggression is mostly verbal e.g., people “shouting” at each other, but also physical, e.g., “fighting”, with some children seeing “a fight every day”. Carers tend to see this as characteristic behaviour for children’s developmental stage and gender, particularly for boys. One mother talked about the “aggression between boys in the sort of pecking order to see who is the toughest”. Three children witnessed severe domestic violence. Low family income appears related to seeing aggression in the community (the street or by neighbours).

Children tend to see more severe forms of aggression, e.g. “stabbing” or “shooting”, in the virtual world. Aggression is present in seemingly age-appropriate programmes and games, i.e., those recommended by rating boards as suitable for the child’s age or broadcasted before ‘the watershed’ (e.g., cartoons). Children also see aggression on inappropriate media, e.g. “shooting games” rated suitable only for those over 18 years, e.g., a 12-year-old boy described playing *Grand Theft Auto* (18+): “you can go round shooting people for no reason”.

Children sometimes have access to age-inappropriate media accidentally, e.g., free games with a purchased game console, or intentionally, sometimes against parental rules, e.g. at friends’ houses. Carers talked about a generation gap, remembering growing up with less, or a “different breed” of, virtual aggression (e.g., Tom and Jerry). Carers are sometimes unaware of the aggressive content of video games they buy for their children. Some carers thought it difficult to protect children: aggression “is everywhere” and continuous monitoring of what is watched or played is impossible, although letting children decide may be risky. A few carers were radical, saying video games “should all be taken completely off the shelf so nobody can go on them. They only bring violence.”

Boys, more than girls, are interested in video games depicting aggression. Children and carers perceive boys’ preferences to be related to gender-specific competitiveness: the competitive nature of the games challenge boys to move “on to the next level”, they “love to win” and “aggression is the excitement”. Virtual settings also permit things impossible in real-life “because you’d just get arrested”. Children and carers talked about “peer pressure”, “like a stigma” and “getting picked on at school” for not playing such games. Society and the media market, accessibility and appeal of games and the lack of exciting but non-aggressive games, fathers’ and peers’ similar preferences, and lack of outdoor activities, are also believed to influence boys’ preferences. Parental restrictions sometimes have the opposite effect: boys play forbidden games more.

### Perspectives of children and carers

**Figure 2:**
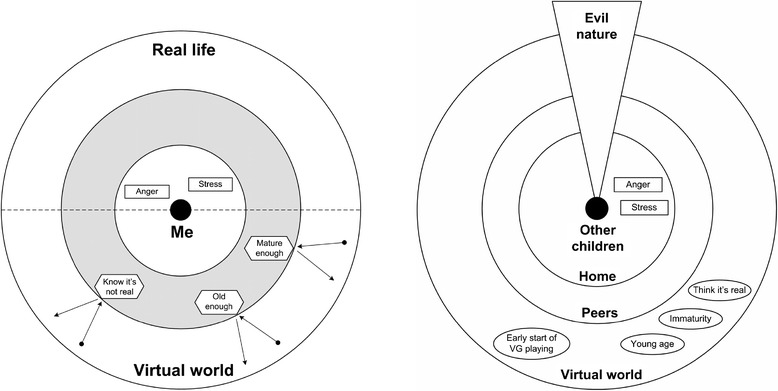
**Child model:** ***Others but not me.*** Contributing factors to children’s aggression. Protective factors against influences of aggression seen in the virtual world. Risk factors related to influences of aggression seen in the virtual world.

**Figure 3:**
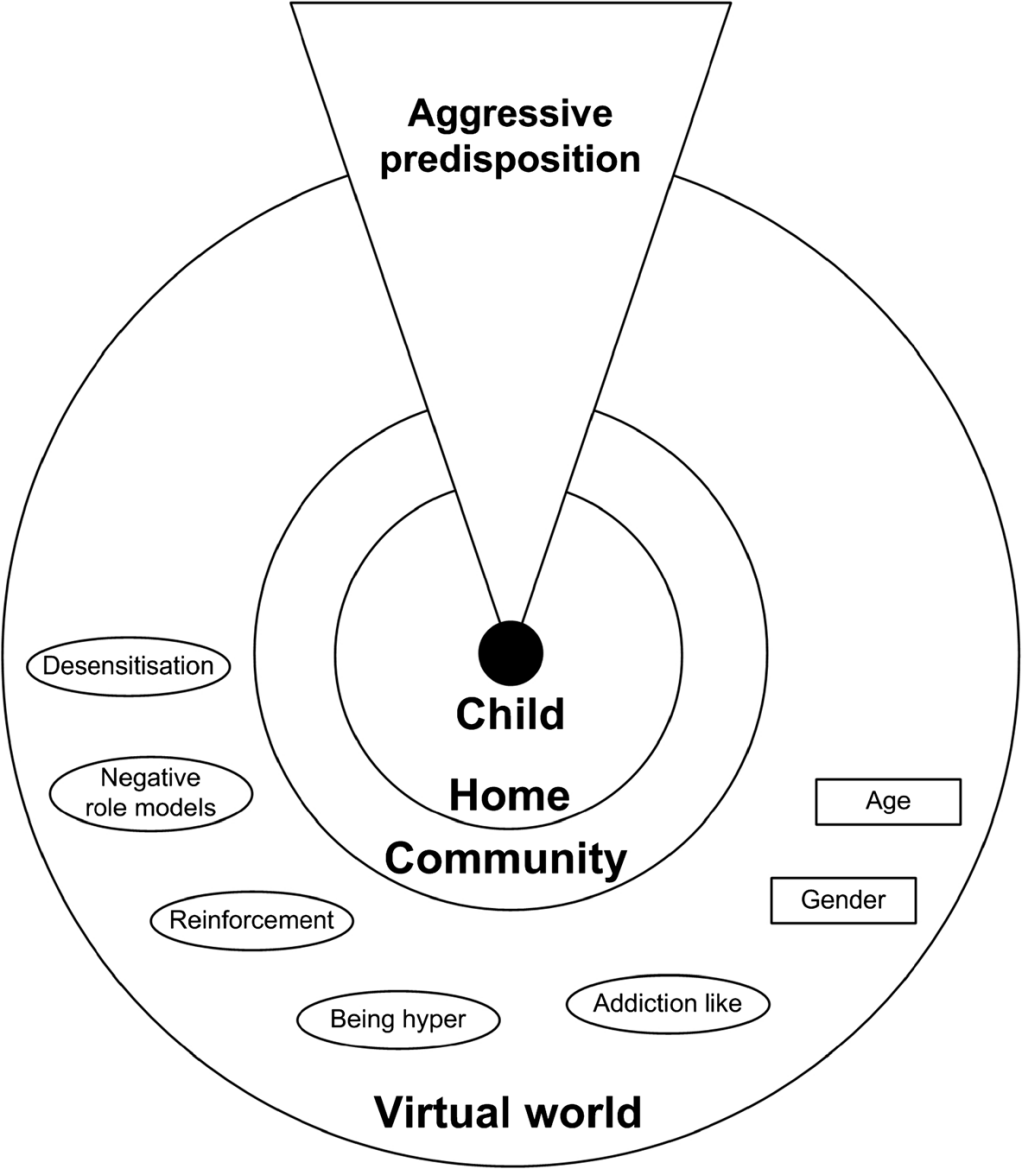
**Carer model:** ***Nature and nurture.*** Factors related to influences of aggression seen in the virtual world. Possible explanations for influences of aggression seen in the virtual world.

Children and carer views on associations between aggression seen and exhibited aggression inform two distinct models of thinking: the child model of *others but not me* (Figure 2) and the carer model of *nature and nurture* (Figure 3). Children thought they could be “aggressive” when “angry” or “stressed” rather than because of seeing aggression. The virtual world is clearly separated from real-life: children feel scared or upset by aggression seen “for real” when they empathise with someone being “hurt”, but have neutral feelings towards aggression seen in TV programmes or video games, even if severe. They feel the latter is “just a game” or “just a cartoon” and “not hurting anyone”; carers were aware of this attitude: “He understands they are make-believe… [he thinks] they don’t affect him.” Most children see themselves as being at an age or developmental stage where they have learned to differentiate reality from fiction and to understand the potential consequences of aggression.

Children distinguish between non-realistic/cartoon-like and realistic/human-like aggression. The first typically involves “goodies” fighting “baddies” whose “bodies disappear”, thus being “not really violent” and “funny”. Examples include The Simpsons or Lego games. “Too graphic” media depicting “proper human” people that “move the way they’d get shot in real-life” or “body parts” are considered “realistic” and “violent”; a video game is “violent” only if they “see any blood” in it, according to some children.

Children and carers shared views on other people’s aggression. ‘Other’ children may behave aggressively because they are “mean” or “evil”, angry or stressed, were “taught the wrong way by their parents”, or because of parental separation, domestic violence or physical abuse. Children provoked by others exemplified peer influence. Most children thought “really young people”, who cannot distinguish between reality and fiction, could “imitate” their favourite virtual “hero”. Significance was placed on early exposure (3-4 year olds) to aggression, e.g., a 10-year-old boy said, “Little children tend to copy what they see on TV or play. So if they pick up violent stuff, they play violent games, it’s more likely they’re gonna grow up to be like that”. One child noted a positive, protective aspect of virtual aggression: “it’s got to have violence because then you can see how bad it actually is and the effects of it so then people wouldn’t do it”.

Carers thought of aggression as a combination of inner and environmental factors. Inner factors include an “aggressive predisposition”, “something inbuilt” in a child’s “genes” or “mental make-up”. Outside influences, or “nurture”, include, more importantly, real-life and the virtual world. The latter was considered to add to children’s predispositions, pre-existing behavioural problems and real-life aggression: “in a child that was already going to be aggressive it might exacerbate it, but I don’t think it would cause it alone in a child who wasn’t aggressive.” One mother prevented her son from watching certain TV programmes because “there’s anger inside him anyway”, not wanting to “feed that anger”. Most carers emphasised the vital role of family and community in helping children use the virtual world “within a controlled environment”, i.e., to explain that aggression “isn’t a good act”, and the nature and consequences of aggression, thus limiting its behavioural influences.

Carers felt the distinction between reality and fiction sometimes becomes blurred, especially when aggression is depicted without its “negative side”. One mother noted the way her son “thinks ‘if Bart can do it, I can do it.’ Bart Simpson is a real person to him.” Another mother had prevented her 9-year old son from playing an aggressive game: “He says he can distinguish between what’s real and what’s not real but what’s not real is coming into the real world in the form of his aggression”. Carers also distinguished between non-realistic/cartoon-like and realistic/human-like aggression, the latter possibly having stronger behavioural effects. Both children and carers see aggression and violence as distinct: violence is “physically doing something” to another person, thus at the more severe end of the aggression spectrum.

Carers’ explanations for any behavioural influences of virtual aggression included “desensitisation” (becoming used to aggression and think “it’s the norm”); provision of “role models” to be “copied”; and “mirroring” or reinforcement of real-life aggression. “Addiction” and gender were raised by two mothers, whose sons’ aggression was seemingly caused by attempts to interfere with their “obsession” with playing video games. This behaviour was compared to that of a “drug addict”, linked, in part, to boys’ competitiveness. Video game playing and sometimes watching TV, regardless of content, were said to make some children “hyper”, “their brains on the go all the time”, contributing to aggression, especially in boys.

Carers also discussed the potential role of age and developmental stage (see above). Older children “already developed their own sets of morals and values” and seeing aggression “wouldn’t affect them so much”, although the “impressionable” teenage stage was mentioned.

## Discussion

To our knowledge, this study is the first to report on aggression, seen or exhibited, in a UK-based sample of children with BED attending CAMHS. Our sample’s mean scores on most CAS-P subscales were higher than those for children with oppositional defiant disorder, but below the means for children with conduct disorder on all subscales, in an American clinic-based sample of children of similar age [16]. We found similar low frequencies of weapon use, perhaps related to the young age of the sample.

Our findings of multiple real and virtual sources of seeing aggression, with severe forms seen more often in the latter, agree with earlier Israeli research with primary school children [22]. Our sample’s reports of seeing aggression on TV, including age-inappropriate programmes, contradict Lowdermilk’s findings that American, primary school children with BED reported mainly watching positive, family-friendly programmes to escape the reality of their sometimes violent home lives [10].

Our findings that children of this age/ developmental stage make a clear distinction between, and appear to have different emotional responses to real-life and fictional aggression, with potentially different behavioural consequences cohere with recent research in adults. Ramos and colleagues’ findings support the key role of the fantasy-reality distinction in mediating viewers’ cognitive and emotional processing of violence, and go against the idea of desensitisation i.e., media violence does not necessarily reduce viewers’ empathy towards real-life violence [23]. Younger children’s potential lack of ability to distinguish between the fictional and the real, mentioned by the children in this study, was also discussed by Byron [8]. This has been disputed, however, by other researchers who argued that children start to develop the ability to use context to make the fantasy-reality distinction between the ages of 3 and 5 years [24].

Byron also found that children often talk about playing 18-rated games [8]. Her findings re-iterate ours on children’s notion of “it’s only a game”; parents’ lack of awareness of game content; parental concerns about desensitisation and risk of addiction; and the relevance of a child’s individuality. Her similar findings on parental concerns over children getting more access to video games surreptitiously if playing is restricted at home parallel the “forbidden-fruit effect” described by Bijvank and colleagues: restricting young people’s access to video games by using age and violent-content labels may increase their attractiveness [25].

Our finding that children of this generation are more familiar with video games than their carers echoes Hulme’s concept of “digital natives”: those growing-up with new information and communication technologies are fundamentally different from previous generations in the way they communicate, seek information, interact and entertain themselves [26]. They may watch TV or play video games when lacking alternative, e.g. outdoor activities [27], perhaps explaining in part carers’ difficulties in controlling children’s access to the virtual world. Generational differences in video game experience may have contributed to the observed distinction between children’s and carers’ views on the behavioural impact of violent games [28].

Our results suggest that aggression results from a combination of inner and environmental factors, where family and community have a key role in limiting the influence of aggression on children’s behaviour. This coheres with multiple risk factor models of aggression and the ecological model of child development, which integrates individual, family and environmental factors [4,29]. Carers’ own explanations for any association between aggression seen in the media and children’s aggression (aggressive predisposition, “copying” negative role models, reinforcement of real-life aggression, desensitisation, being “hyper” after watching TV or playing video games) appear to be consistent with theories such as social learning theory, the cognitive neo-association model, social information-processing model and arousal theory [30-33]. But their suggested role of virtual aggression i.e., only additional to children’s aggressive predisposition, pre-existing behavioural problems and secondary to aggression seen in real-life, coheres with the more recently proposed “catalyst” model [34]. This model suggests that severe forms of aggression results from a combination of genetic and proximal environmental influences (family and peers), with distal environmental factors such as the media having a less important role of modelling the form of aggressive behaviour.

Recent research has also focused on understanding individuals’ motivations for video game play, including violent games, and placing it within the context of normal development [35]. Our findings on reasons for children’s, particularly boys’ interest in aggressive video games (competition, challenge, fun, excitement, doing things they cannot do in real-life, peers’ preferences, lack of other activities) cohere with studies on motivations for, and experiences of video game play among children [35] and adults [36], and the motivational model of video game play [37]. They are also consistent with Adachi and Willoughby’s findings on the competitive nature of video games as a third variable in the observed longitudinal association between violent video game play and aggression [38].

Our study augments findings of a systematic review that reported differences between children’s and parents’ views on whether seeing aggression on TV affects children; and possible associations between watching TV, regardless of aggressive content, and children’s aggression [10]. The way children perceive cartoon-like aggression as “not really violent” and “funny” could partly explain the contradictory results of earlier experimental studies investigating the effects of watching aggressive cartoons on children’s behaviour.

Our quantitative study is limited by the small size and gender and ethnic imbalance of the sample, potentially affecting its generalizability. The qualitative findings also may be less representative of the views of children and carers of other than White British ethnicity. The qualitative data analysis was informed by the researchers’ experiences as mental health professionals and *a priori* reasoning about possible links between seen and exhibited aggression.

The low recruitment rate reflects challenges in researching this doubly *hard to reach* population: recruiting children in mental health contexts. Identified barriers were related to: participants, e.g., the children’s complex mental health problems and subsequent burden on families; the research topic (sensitive, raising ethical dilemmas); and mental health services, e.g., clinicians acting as *over-zealous gatekeepers* [39], restricting access to families. Nonetheless, there were no significant differences between participants and non-respondents/those opting-out, on child’s age, gender and referral reasons.

There is yet no definitive evidence for or against a direct relationship between aggression seen in the media and children’s aggression. Our study indicates that future research in this field should take a broader, ecological perspective, investigating individual, developmental, family and environmental factors. Virtual aggression seems to play a secondary role to real-life aggression, hence validated measures of aggression in both contexts, distinguishing between types of aggression (verbal and physical) are still needed. The potential role of gender and aggressive predisposition, as mediating or moderating factors in any observed relationship between exposure to media violence and aggression needs nuanced investigation. Third variables that may be key, and need to be further operationalized in terms of research methods, are the abilities to distinguish reality from fantasy, and realistic from non-realistic fiction, and competitiveness. Several other factors should be controlled for in future studies: peer relationships, family income, type and size of household, and parental formal education level. A child’s cognitive, social and emotional developmental stage may be more significant than chronological age. Further research among children with pre-existing mental health problems is warranted, in view of ongoing debate regarding their potential vulnerability [40].

Our quantitative and qualitative findings indicate that when seeking subjective data from informants, complementarity of child/parent reports rather than inter-rater reliability is worth seeking, as child and adult perspectives potentially uncover different underlying phenomena. Example are children’s distinction between real-life and fictional aggression, and their views that the depiction of blood differentiates between what is and what is not violent.

## Conclusion

Children, regardless of their socio-demographic background or aggressive behaviour levels, see a lot of aggression in many parts of their lives. Future research may either confirm or refute the existence of an association between media violence and children’s aggression. Until then, clinicians, professional organizations and policy-makers should address aggression coming into children’s lives through all relevant means, primarily real-life and secondly virtual. For example, in clinical contexts, children are often asked about experiences of abuse but much less about seeing aggression at home, at school, in the neighbourhood or in the media. Children’s access to programmes and games should be supervised [35] and accompanied by developmentally appropriate discussion about the aggression seen and its potential real-life consequences. Carers should be careful, particularly when a child has an aggressive predisposition and at an earlier developmental stage, as aggression seen could have greater impact on these children.
